# Long noncoding RNA HOTAIR promotes metastasis of renal cell carcinoma by up-regulating histone H3K27 demethylase JMJD3

**DOI:** 10.18632/oncotarget.15047

**Published:** 2017-02-03

**Authors:** Ming Xia, Lv Yao, Qiaoxia Zhang, Feng Wang, Hongbin Mei, Xiaoqiang Guo, Weiren Huang

**Affiliations:** ^1^ Department of Urology, Shenzhen Second People's Hospital, The First Affliated Hospital of Shenzhen University, Shenzhen 518035, Guangdong, China; ^2^ State Engineering Laboratory of Medical Key Technologies Application of Synthetic Biology, Key Laboratory of Medical Reprogramming Technology, Shenzhen Second People's Hospital, The First Affliated Hospital of Shenzhen University, Shenzhen 518035, Guangdong, China; ^3^ Laboratory of Molecular Iron Metabolism, College of Life Science, Hebei Normal University, Shijiazhuang 050024, Hebei, China; ^4^ Department of Urology, Peking University Shenzhen Hospital, Institute of Urology of Shenzhen PKU–HKUST Medical Center, Shenzhen, 518036, Guangdong, China; ^5^ Department of Urology, The Third Affiliated Hospital of Southern Medical University, Guangzhou 510000, Guangdong, China

**Keywords:** long noncoding RNA, HOTAIR, renal cell carcinoma, histone demethylase, JMJD3

## Abstract

Long Noncoding RNAs (lncRNAs) are a kind of non-protein coding transcripts longer than 200 nucleotides, and play important roles in diverse biological processes, such as embryonic development and apoptosis. Homeobox (HOX) transcript antisense intergenic RNA (HOTAIR) is a negative prognostic factor in a variety of human cancers, such as breast, liver and lung cancers. HOTAIR can promote cancer cell metastasis by reprogramming chromatin organization. In the present study, HOTAIR expression was elevated in tissues of renal cell carcinoma compared to adjacent normal tissues, and positively correlated with metastasis (P<0.05). The cell migration was inhibited in scratch test and transwell assay after HOTAIR knockdown (P<0.05). Further researches revealed that histone demethylase JMJD3 was reduced and its target gene *Snai1* expression was down-regulated after HOTAIR suppression (P<0.05). Meanwhile, the level of histone methytransferase EZH2 target gene *PCDHB5* was increased (P<0.05). Collectively, these data suggest that HOTAIR is an important promoter in metastasis of renal cell carcinoma and also plays a dual regulatory role in chromatin state by effecting both histone metylation and demethylation at different gene loci.

## INTRODUCTION

Renal cell carcinomas (RCC) is among the 10 most common cancers, which accounts for 2% to 3% of all adult malignancies and causes 100,000 deaths per year worldwide [1]. Distant metastases, the major cause of poor prognosis, are present at the time of initial diagnosis in approximately one third of RCC patients [2]. Average survival time for the metastatic RCC was under a year. So, it is essential for improvement of metastatic RCC therapy to further understand the mechanism of RCC.

Long noncoding RNAs (lncRNAs) are non-protein coding transcripts longer than 200 nucleotides, and important regulators in gene expression. lncRNAs have gained widespread attention as a potentially new and crucial player of biological regulation [3]. Many studies have demonstrated that abnormal content or function of lncRNAs are related to tumor formation, progression, and metastasis of many kinds of cancer, such as prostate, bladder, and kidney cancers [4]. Therefore, it is very important for clinical diagnosis and therapy of diverse cancers to explore the biological function of lncRNAs [5].

HOTAIR (Homeobox transcript antisense intergenic RNA) is one kind of lncRNAs, which has 2,158 bases and is localized to a boundary in the HOXC gene cluster [6]. HOTAIR is overexpressed in breast, colon and liver cancers, and plays important roles in cancer invasion and metastasis [7]. HOTAIR is also a negative prognostic factor in many cancers [8]. Many lncRNAs can regulate chromatin states and play biological roles by epigenetic modification [9]. HOTAIR can interact with the polycomb repressive complex 2 (PRC2) (including histone methyltranferas EZH2 and other factor SUZ12 and EED) to enhance H3K27 trimethylation and decrease expression of multiple genes [10]. It also indicated that HOTAIR plays a critical role in cancer metastasis by effect genome-wide reprogramming of PRC2 [11]. All these studies elucidated its biological role in cancer development and the potential molecular mechanism. However, the functional impact of HOTAIR in RCC remains to be clarified and other epigenetic effect of HOTAIR is less understood.

In this study, our results show that the expression of HOTAIR is higher in cancer tissues than corresponding noncancerous tissues of RCC patients, and correlated tightly with metastasis. It is also indicated that HOTAIR promotes cell migration and invasion. Furthermore, HOTAIR not only affects the activity of histone H3K27 methyltransferase EZH2, but also regulates the role of histone H3K27 demethylase JMJD3. So, we propose that HOTAIR is a dual epigenetic regulator, which leads to different H3K27 methylation status at different gene loci.

## RESULTS

### The expression of HOTAIR is positively correlated with metastasis of RCC

**Figure 1:**
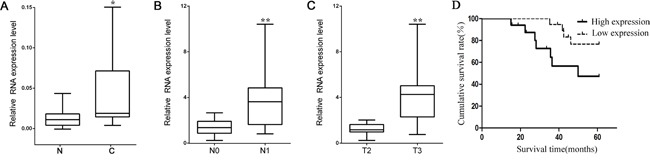
*HOTAIR* is overexpressed in kidney cancer **A**. *HOTAIR* expression was higher in cancer tissues (C) than noncancerous tissues (N) from RCC patients (n = 36). **B**. The expression of *HOTAIR* was higher in RCC patients with tumors already spread to regional lymph nodes (N1, n=15) than those with tumor localized in the kidney (N0, n=21) **C**. The expression of *HOTAIR* was also higher in RCC patients with tumors extending beyond the kidney (T3, n=10) than those with tumor detected only in the kidney (T2, n=26). **D**. The cumulative survival rate was significantly higher in the low HOTAIR expression group than that in the high HOTAIR expression group (P=0.04).*, *P* < 0.05; **, *P* < 0.01.

To understand the biological significance of lncRNA HOTAIR in RCC development, the mRNA levels of HOTAIR were examined in cancer tissues and corresponding noncancerous tissues from 36 RCC patients using qRT-PCR. HOTAIR was highly expressed in cancer tissues than noncancerous tissues (P < 0.05, Figure 1A). The expressions of HOTAIR were higher in tumors spread to regional lymph nodes (N1) compared with tumors localized only in the kidney (N0) (P < 0.01, Figure 1B). The transcription level of HOTAIR was higher in tumors extending into major veins or perinephric tissues (T3) than in tumors limited to the kidney (T2) (P < 0.01, Figure 1C). Kaplan-Meier analysis indicated that the prognosis was poorer in RCC patients with high HOTAIR expression than those with low expression (Figure 1D). All these data indicated that HOTAIR is an important promoting factor in RCC development and maybe related to metastasis of RCC.

### HOTAIR promotes kidney cancer cell migration

**Figure 2:**
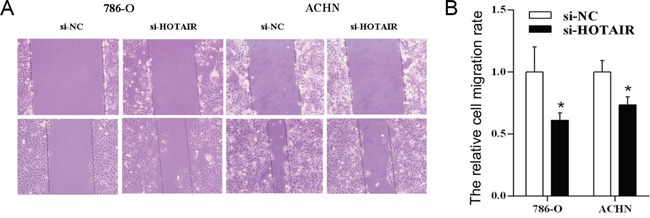
The knockdown of *HOTAIR* inhibits cell migration 786-O and ACHN cells were treated with si-HOTAIR, and the effects on cell migration were determined with cell scratch test. **A**. The cell migration of both cell lines. **B**. Quantitative results. Values are the mean of triplicate samples from a representative experiment. *, *P* < 0.05.

To understand the effect of HOTAIR on cell migration, the cell scratch test was followed. The cell migration was significantly inhibited in kidney cell lines 786-0 and ACHN after knockdown of HOTAIR (si-HOTAIR) by RNA interference (Figure 2A). And the difference is obvious between HOTAIR knockdown (si-HOTAIR) and control (si-NC) (*P* < 0.05, Figure 2B).

### HOTAIR increases kidney cancer cell invasion

**Figure 3:**
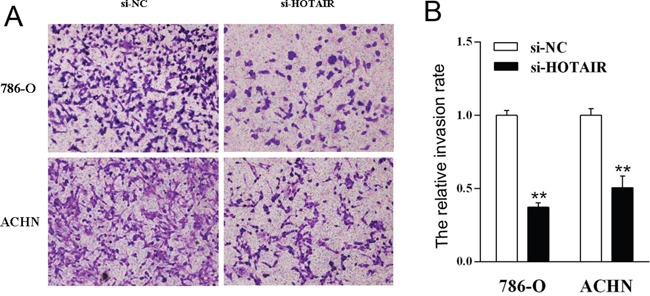
The knockdown of *HOTAIR* inhibits cell invasion 786-O and ACHN cells were treated with si-HOTAIR, and the effects on cell invasion were determined with cell transwell test. **A**. The cell invasion of both cell lines. **B**. Quantitative results. Values are the mean of triplicate samples from a representative experiment. **, *P* < 0.01.

To further explore the role of HOTAIR on cell invasion, the cell transwell assay was carried out. The capacity of cell invasion was obviously reduced in kidney cell lines 786-0 and ACHN after HOTAIR suppression (Figure 3A). There is significant difference between HOTAIR suppression (si-HOTAIR) and normal (si-NC) (P < 0.01, Figure 3B). All these results showed that HOTAIR could increase kidney cancer cell migration and invasion *in vitro*.

### HOTAIR can affect histone H3K27 methylation and its target genes’ expression

**Figure 4:**
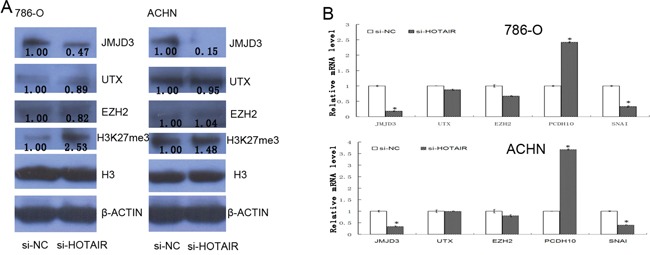
HOTAIR promotes *SNAI1* expression by effecting histone demethylase JMJD3 **A**. The cell extracts were prepared from 786-0 and ACHN cells and examined by Western blotting after *HOTAIR knockdown*. **B**. The total RNA was extractedand mRNAs of several genes in 786-0 and ACHN cells were quantified by qRT-PCR. Values are the mean of triplicate samples from a representative experiment. *, *P* < 0.05.

To elucidate the potential mechanism of HOTAIR, H3K27 methylation modifying enzymes were studied. At protein level, histone H3K27 dimethylase JMJD3 is significantly decreased in 786-0 and ACHN cells after HOTAIR knockdown, and the content of H3K27me3 is increased in 786-0 cells while it is not obviously changed in ACHN cells (Figure 4A). At mRNA level, there is a decrease of *JMJD3 in both* 786-0 and ACHN cells, whereas no differences of *EZH2* and *UTX* were observed, after HOTAIR knockdown (P < 0.05, Figure 4B). Furthermore, the *PCDH10* expression (regulated by EZH2) is upreguled and *SNAI1* (regulated by JMJD3) is downregulated (regulated by JMJD3) after HOTAIR knockdown (P < 0.05, Figure 4B). So, HOTAIR can affect the function of histone H3K27 demethylase JMJD3.

## DISCUSSION

LncRNAs are emerging as key players in various fundamental biological processes and altered expression of them is specifically associated with tumorigenesis, tumor progression and metastasis [12]. A better understanding of association between lncRNAs and tumor biology provides novel therapeutic targets for the treatment of urologic cancers, including kidney cancer [13]. Many studies have demonstrated that HOTAIR is overexpressed in many cancers, such as breast, gastric and colorectal cancers [14–16]. Our results indicated that HOTAIR was highly expressed in cancer tissues than in noncancerous tissues. Our previous researches have indicated that HOTAIR can promote cell proliferation and inhibit apoptosis of renal cancer cell lines 786-O and ACHN [18]. It was also showed that HOTAIR is a negative factor of prognosis and positively correlated with metastasis [17]. Our study further proved this conclusion in RCC.

HOTAIR can be also regarded as an oncogene and promotes cancer metastasis [19]. In kidney cancer cells 786-0 and ACHN, HOTAIR significantly increases the abilities of cell migration and invasion. These results were consistent with previous studies.

HOTAIR can bind the PRC2 subunit EZH2 and coordinate PRC2-dependent epigenetic regulation to inhibit gene expression [20]. HOTAIR regulates many genes silencing including *PCDH10* and *HOXD10*. *PCDH10* is a tumor suppressor gene and frequently inactivated in many cancers including RCC [21]. PCDH10 suppresses metastasis and its genetic loss predicts adverse prognosis [22]. In present study, the expression of *PCDH10* was re-activated after *HOTAIR* knockdown, which is responsible for decreasing cell migration and invasion. So, our results proved that HORAIR exhibits pro-oncogenic activity partly by regulating PRC2-mediated *PCDH10* gene silencing.

JMJD3 is a histone demethylase and can activate target genes expression by catalyzing H3K27me2/3 demethylation [23]. *JMJD3* is also overexpressed in various cancers [24]. Our previous researches indicated that JMJD3 is highly expressed in cancer tissues than noncancerous tissues in RCC [25]. Other studies demonstrated that JMJD3 can promote transcription of the metastasis-related gene *SNAI1* by removing H3K27me3 at the promoter of *SNAI1*, which is important for epithelial-mesenchymal transition (EMT)[26]. SNAI1 expression is a potential adverse prognostic biomarker for survival of patients with RCC [27]. Because JMJD3 is a positive regulating factor of cell migration and invasion, and HOTAIR has also the activity to promote EMT and metastasis [27], it is possible that the biological effects of HOTAIR are mediated by JMJD3. In the study, both of JMJD3 mRNA and protein were decreased after HOTAIR knockdown, and the expression of *SNAI1* was accordingly downregulated. It means that HOTAIR is involved in tumor metastasis of RCC partly through increasing the JMJD3-mediated *SNAI1* upregulation. Latest research shows that HOTAIR plays a crucial role in the Snai1-mediated EMT [29], which is consistent with our results.

**Figure 5:**
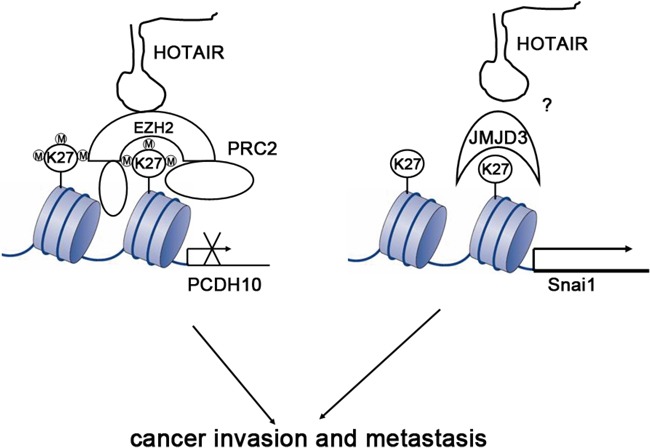
Proposed mechanism of HOTAIR action HOTAIR is a dual regulator of histone state. HOTAIR can interact with PRC2 (EZH2 and other factor) and lead to H3K27 trimethylation in some gene loci as scaffolds, which is important to suppress expression of many genes including *PCDH10*. HOTAIR also can influence the content of histone demethylase JMJD3 and cause H3K27 hypomethylation in other gene loci by activating transcription of several genes including *SNAI1*.

Based on the results, a mechanism of HOTAIR action was proposed (Figure 5). HOTAIR can serve as a scaffold of H3K27me3 PRC2, which inhibits the expression of related target genes such as *PCDH10*. Simultaneously, HOTAIR is also a regulator of histone demethylase JMJD3, which promotes epithelial-mesenchymal transition though upregulation of *Snail*.

In conclusion, our work illustrates the biological role and potential mechanism of HOTAIR in RCC metastasis. We show that HOTAIR is a negative prognostic factor for RCC metastasis, and HOTAIR knockdown can inhibit cell migration and invasion. HOTAIR is a dual player in histone methylation state, both H3K27 methylation and demethylation, at different gene loci. Further researches should be done to explore the regulatory role of HOTAIT on JMJD3 expression.

## MATERIALS AND METHODS

### Patients and tissue specimens

All the cancer tissue and corresponding noncancerous tissue samples of 36 RCC patients were provided by the Biobank of Complex Diseases in Shenzhen between 2010 and 2012 in China. All the tissues were obtained according to a protocol approved by Ethics Committee Review Broad of Shenzhen Second People's Hospital. All patients gave signed informed consents. All resection samples were confirmed to be RCC by clinical pathology.

### Cell culture and transfection

The human kidney cancer cells 786-O and ACHN were purchased from cell resource center of Shanghai Institutes for Biological Sciences, Chinese Academy of Science. Both cells were maintained in DMEM (GIBCO, Grand Island, USA) supplemented with 10% heated-inactivated fetal bovine serum (FBS, Hyclone, Logan, USA). Cells were cultured at 37° with 5% CO_2_. For RNA interference assay, 786-O and ACHN cells were seeded at 70-80% confluency before transfection and transfected with siRNAs using Lipofectamine 2000 (Invitrogen, Carlsbad, CA, USA) according to the manufacturer's protocol. The effect of HOTAIR was evaluated with qPCR. All the siRNAs (20nM) were synthesized by Genepharma (Shanghai, China) and the sequences were as follows: negative control (si-NC): 5′-UUCUCCGAACGUGUCACGU-3′; HOTAIR (si-HOTAIR): 5′-UUAAGUCUAGGAAUCAGCACGAAGC-3′.

### Cell migration assay (cell scratch test)

Both 786-0 and ACHN cells were seeded on the 6-well plates and transfected with si-HOTAIR or si-NC respectively. At 6h post-transfection, a clean line was created with a sterile 200μl pipette tip. The migration of cells was monitored using a digital camera system and imaged at the time of 0h and 24h. The relative cell migration rate was calculated.

### Cell invasion assay (cell transwell test)

At 24 hours post-transfection, 1×10^5^ cells with 100 μl serum-free medium were plated into the upper chambers (24-well insert, pore size 8 μm, Corning) which were added with Matrigel (1:8, 50μl/well, BD Bioscience, San Jose, CA, USA). Concurrently, the lower chambers were filled with 500 μl medium containing 10% fetal bovine serum. Cells were cultured at 37°C in a 5% CO_2_ atmosphere for 48 hours. Cells under the surface of the lower chamber were washed with 1×PBS, fixed with 4% paraformaldehyde for 20 min, stained with 0.1% crystal violet for 25 min, and then washed 3 times. Invaded cells were observed under the inverted microscope and imaged. Afterwards, each chamber with the invaded cells was soaked into 1ml 33% acetic acid for 10 min to wash out the crystal violet. 100μl/well 33% acetic acid were added into 96-well plates, and the absorbance was measured at a wavelength of 570nm using a microplate reader (Bio-Rad, Hercules, CA, USA).

### Quantitative real-time polymerase chain reaction (qRT-PCR)

Total RNAs were prepared from tissues or cells using Trizol reagent (Invitrogen) in accordance with manufacturer's instructions. Then, cDNA was synthesized from 1μg of total RNA using a Fermentas RT system (Thermo Scientific, Wilmington, DE, USA) according to the manufacturer's protocol. The cDNA was subjected to qRT-PCR. qRT-PCR was performed in 20 μl reaction mixture containing 10μl of SYBR Premix, 0.5μM of forward and reverse primers, and 1μl template cDNA on LightCycler480 System (Roche, Foster City, CA, USA). The primers were designed according to the human HOTAIR, EZH2, JMJD3, UTX, PCDHB5, SNAI1, 18S rRNA and ACTIN genes sequences reported in GenBank. Relative expression level of HOTAIR was normalized to the internal reference 18SrRNA, and other coding genes levels normalized to ACTIN. The primers were synthesized by Sangon (Shanghai, China) and sequences were listed in Table 1.

**Table 1:** Primers used in the experiment

gene	forward primer	reverse primer
*HOTAIR*	GGTAGAAAAAGCAACCACGAAGC	ACATAAACCTCTGTCTGTGAGTGCC
*JMJD3*	CGCTGCCTCACCCATATCC	ATCCGCGACCTCTGAACTCT
*UTX*	GTGCTCACGCTCGGAGAAA	GTGGGAAACAGCTCGAATGGT
*EZH2*	AGGAGTTTGCTGCTGCTCTCACC	CCCGTTTCAGTCCCTGCTTCCC
*PCDHB5*	AAGGGCATTGGATTTCGAGG	GAGAGCGTAGACATGGTGAGT
*SNAI1*	AGATGAGCATTGGCAGCGAG	TCGGAAGCCTAACTACAGCGA
*18SrRNA*	CGGCGGCTTTGGTGACTCTAG	CCGTTTCTCAGGCTCCCTCTCC
*ACTIN*	CCACTGGCATCGTGATGGACTCC	GCCGTGGTGGTGAAGCTGTAGC

### Western blots

786-0 and ACHN cells were washed with PBS buffer twice and then homogenized in 200μl radioimmuno-precipitation assay (RIPA) buffer containing the protease inhibitors cocktail(1 mmol/L) and phenylmethylsulfonyl fluoride (100μg/mL). Homogenates were centrifuged and supernatants were collected. A total of 50 μg of protein separated by 10% sodiumdodecyl sulfate-polyacrylamide gel electrophoresis (SDS-PAGE) and transferred to polyvinylidene difluoride (PVDF) membranes. The membrane were saturated with 5% skim milk in TBST (50 mM Tris–HCl, 150 mM NaCl, 0.1% Tween-20) for 2h and then incubated with primary antibodies at 4°C overnight. The primary antibodies used in this study included rabbit polyclonal antibodies to UTX (1:1,000, Abcam, Hong Kong, China), JMJD3 (1:1500, Abcam), EZH2 (1:500, Santa Cruz Biotechnology, Hong Kong, China), H3K27me3 (1:1,500, Epigentek, Brooklyn, USA), H3 (1:2,000, Sigma-Aldrich, St Louis, USA) and actin (1:2,500, Sigma, St Louis, USA). The membranes were incubated with HRP-conjugated goat anti-rabbit antibody (1:5,000, Sigma) for 1 h at room temperature and then exposed to enhanced chemiluminescence substrate (Millipore, Rockford, USA), and detection was performed using a film.

### Statistical analysis

All data represent means ± SD from three independent experiments. The differences between two groups including clinical data and cell experiments were analyzed with unpaired two-tailed Student *t* test. Survival curves were analyzed by the Kaplan-Meier method. *P*<0.05 was considered statistically significant.

## Abbreviations

LncRNAs: Long Noncoding RNAs
HOTAIR: Homeobox transcript antisense intergenic RNA
RCC: Renal cell carcinomas
PRC2: polycomb repressive complex 2
EMT: epithelial-mesenchymal transition.
